# Targeting Certain Interleukins as Novel Treatment Options for Liver Fibrosis

**DOI:** 10.3389/fphar.2021.645703

**Published:** 2021-03-24

**Authors:** Su Yeon An, Anca D. Petrescu, Sharon DeMorrow

**Affiliations:** ^1^ Division of Pharmacology and Toxicology, College of Pharmacy, The University of Texas at Austin, Austin, TX, United States; ^2^ Department of Internal Medicine, Dell Medical School, The University of Texas at Austin, Austin, TX, United States; ^3^ Research Division, Central Texas Veterans Healthcare System, Temple, TX, United States

**Keywords:** interleukins, hepatocytes, macrophages, hepatic stellate cells, CD4^+^ T helper cells, liver fibrosis

## Abstract

The liver is a major metabolic organ and an immunologically complex organ. It produces and uses many substances such as acute phase proteins, cytokines, chemokines, and complementary components to maintain the balance between immunity and tolerance. Interleukins are important immune control cytokines, that are produced by many body cells. In liver injury, interleukins are produced in large amount by various cell types, and act as pro-inflammatory (e.g. interleukin (IL)-6, IL-13, IL-17, and IL-33) as well as anti-inflammatory (e.g. IL-10) functions in hepatic cells. Recently, interleukins are regarded as interesting therapeutic targets for the treatment of liver fibrosis patients. Hepatic cells such as hepatocytes, hepatic stellate cells, and hepatic macrophages are involved to the initiation, perpetuation, and resolution of fibrosis. The understanding of the role of interleukins in such cells provides opportunity for the development of therapeutic target drugs. This paper aims to understand the functional roles of interleukins in hepatic and immune cells when the liver is damaged, and suggests the possibility of interleukins as a new treatment target in liver fibrosis.

## Introduction

Fibrosis is the pathological feature and the end result of chronic inflammatory reaction induced by various types of injuries in several organs including skin, kidney, lung, heart, intestine, and liver. Therefore chronic fibroproliferative diseases is a major public health problem (Nanchahal and Hinz, 2016). In the liver, progressive fibrosis caused by injury, inflammation, and extracellular matrix (ECM) accumulation ultimately results in cirrhosis or hepatocellular carcinoma. The only effective treatment for these terminal liver disorders is liver transplantation (Bataller and Brenner, 2005; Bansal et al., 2016). For this reason, treatment at the liver fibrosis stage that prevents the progression of the disease, and that leads to resolution is very important.

The initiation, perpetuation and resolution of liver fibrosis is a complex process of communication between cells receiving a multitude of signals consisting of various growth factors, chemokines and cytokines (Kubes and Jenne, 2018). Interleukins, a cytokine family that include chemokines, interferons, lymphokines, and tumor necrosis factors, were first thought to be expressed in leukocytes alone, but it was later found that about 40 types of interleukins were produced by many other body cells. Interleukins play an essential role in the activation and differentiation of immune cells as well as proliferation, maturity, migration, and adhesion. They can also have pro-inflammatory or anti-inflammatory or both properties (Hammerich and Tacke, 2014).

IL-6 and IL-1 are representative keystone cytokines in liver disease and have both pro and anti-inflammatory properties. IL-6 is a main regulatory factor in processes of wound healing and tissue regeneration related to various liver injures. It was concluded that IL-6/STAT3 signaling prevents cholestasis and liver fibrosis and has role in regulation of hepatocyte and cholangiocyte functions in the model of sclerosing cholangitis (Mair et al., 2010). However, more recent publications reveal negative effects of IL-6 which can directly induce transition of HSCs to myofibroblast-like cells, in hepatic fibrosis (Kagan et al., 2017). Very recent studies on the role of JAK-STAT signaling in hepatocellular carcinoma (HCC) emphasized that members of IL-6 family of cytokines have emerged as important regulatory factors and are considered to be targets for therapeutic intervention (Lokau et al., 2019). IL-1 family consists of 11 cytokines of the innate immune system. These cytokines trigger various immune responses to liver injuries, i.e. IL-1α, β and IL-33 are proinflammatory cytokines; in contrast, IL-1Ra, IL-36Ra, IL-37, and IL-38 have anti-inflammatory responses, while IL-18 was found to be pro- and anti-inflammatory under different circumstances (Barbier et al., 2019; Chan and Schroder, 2020). Additionally, IL-1 cytokines activate mast cells (MCs) to secrete inflammatory mediators (chemokines and cytokines including IL-1, IL-33 and TNF), and this effect can be counteracted by IL-37 (Gallenga et al., 2019). Thus, it has been determined that IL-37 is an anti-inflammatory cytokine that interacts with IL-18Ra chain and decreases the inflammatory effects of IL-1 cytokines (Toniato et al., 2017; Caraffa et al., 2019; Gugliandolo et al., 2019). Cytokines from IL-36 subfamily of IL-1 are produced by activated MCs and have strong pro-inflammatory effects which can be balanced by IL-38 which binds to IL-36R inducing anti-inflammatory activity (Varvara et al., 2018; Gallenga et al., 2019). Numerous studies have investigated the role of each cytokine and receptor from this family in various types of liver injuries, and have been reviewed in detail recently (He et al., 2021).

This review presents and emphasizes certain interleukins as potential therapeutic targets for liver fibrosis. We specifically focused on the pro-inflammatory interleukins: interleukin (IL)-13, IL-17, and IL-33, anti-inflammatory interleukin: IL-10 based on the numerous studies published in recent years that clearly showed their functions and effects. Furthermore, this review summarizes the origin, function, and signal pathway of these selected interleukins and, also shows the functions of these specific interleukins in hepatocytes, hepatic stellate cells (HSCs), and hepatic macrophages which are main players in liver fibrosis.

## CD4^+^ T Helper Cells

The innate and adaptive immune responses occurring simultaneously or sequentially in time, are initiated by Kupffer cells that recognize liver damage. Following liver injury, inflammatory lymphocytes infiltrate the damaged hepatic parenchyma (Dong et al., 2019; Li et al., 2019). T lymphocytes, which are activated through antigen-specific manner of dendritic cells, help the B lymphocytes to create antigen-specific antibodies, or to remove infected cells. Specially, CD4^+^ T helper cells (CD4 T cells) serve as the total commander of immunity. CD4 T cells can be classified into at least four subsets, referred to as T helper (Th) cells type 1, Th2, Th17, and induced regulatory T cells (Tregs). These cells play a major role in mediating immune response through the secretion of specific cytokines (Luckheeram et al., 2012).

Th1 cells play a particularly important role in mediating immune responses against intracellular pathogens, resistance to mycobacterial infections, and induction of some autoimmune diseases. Their principal cytokine products are interferon gamma, lymphotoxin α, and IL-2 (Paul and Seder, 1994). Th2 cells mediate immune response to extracellular parasites, including helminths, and play an important role in induction and persistence of asthma and other allergic diseases. Th2 cells produce IL-4, IL-5, IL-9, IL-13, IL-10, IL-25, and amphiregulin (Luckheeram et al., 2012). Th17 cells are responsible for mediating immune responses against extracellular fungi and bacteria, and for inducing the many organ-specific autoimmune diseases. Th17 cells produce IL-17, IL-17f, IL-21, and IL-22 (Zhu and Paul, 2008). IL-17 leads to the induction of pro-inflammatory cytokines, including IL-6, IL-1, tumor necrosis factor-α (TNF-α), and thus has an important role in inducing inflammatory responses (Luckheeram et al., 2012). Tregs play a critical role in the maintenance of self-tolerance to self and foreign antigen. Increasing Tregs numbers and/or enhancing their suppressive function may be beneficial for preventing allograft rejection and treating autoimmune diseases. The main effector cytokines of Tregs include IL-10, transforming growth factor-β (TGF-β), and IL-35 (Zhu and Paul, 2008; Luckheeram et al., 2012).

CD4 T cells release a wide range of inflammatory intermediates, particularly interleukins, which can act as the pro-inflammatory, anti-inflammatory, or two functions in liver fibrosis (Hammerich and Tacke, 2014; Sziksz et al., 2020). However, selecting interleukins with conflicting functions depending on the stage of liver disease as a therapeutic target requires more attention in the treatment process. In the case of IL-6 or IL-22, it prevents fibrogenesis but promotes hepatocellular carcinoma, so the risk of tumor occurrence should be considered in the treatment of patients with hepatic fibrosis (Schmidt-Arras and Rose-John, 2016; Wu et al., 2020). Therefore, it is very important to select and study targets capable of blocking pro-inflammatory (IL-13, IL-17, and IL-33) or inducing anti-inflammatory interleukin (IL-10) in the complex process of liver fibrosis.

## Target Interleukins

### Interleukin-10

**FIGURE 1:**
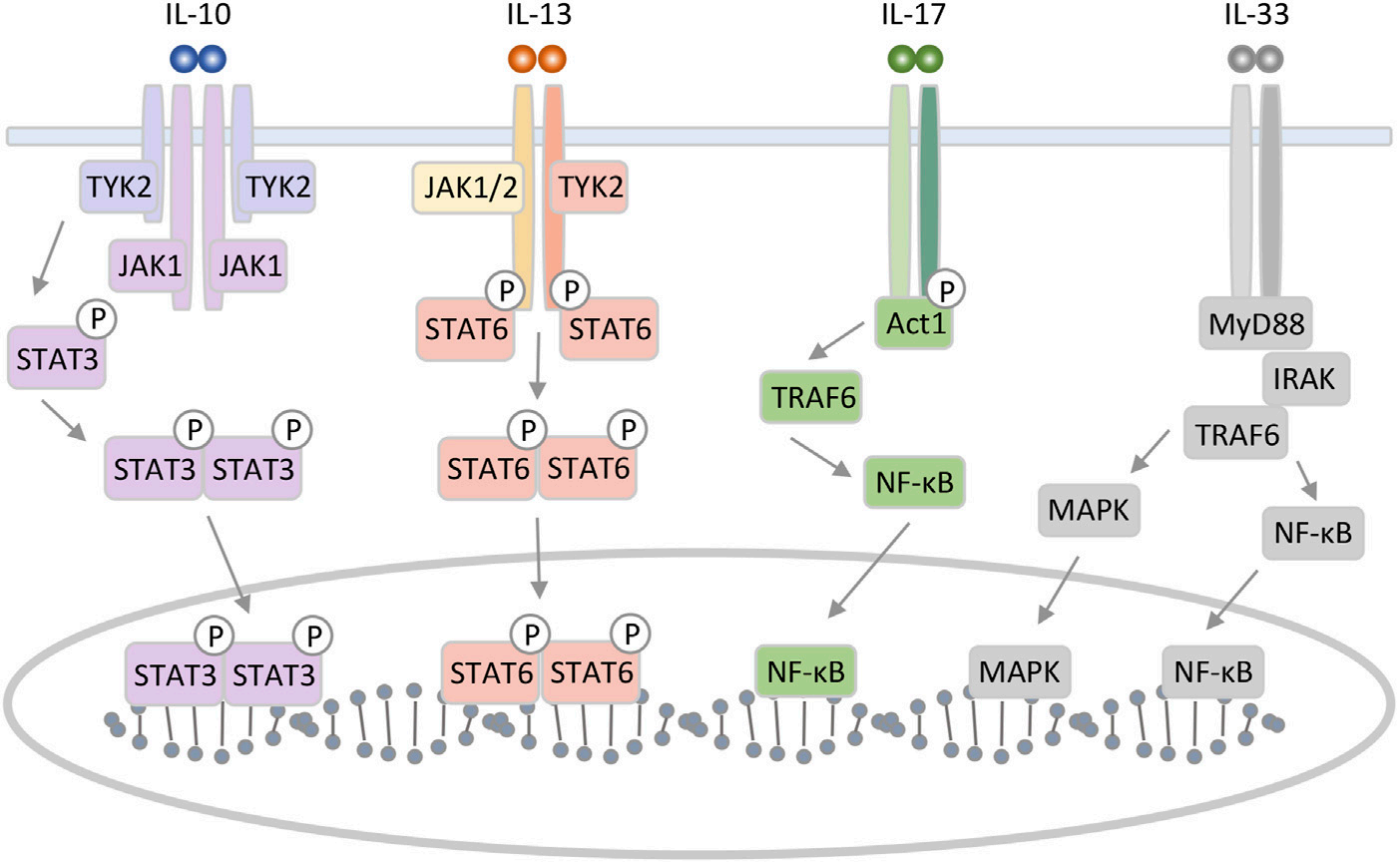
Schematic representation of IL-10, 13, 17, and 33 interaction mechanism with their specific receptors. IL-10 can form a homodimer that binds to the tetrameric heterodimer IL-10 receptor and initiates an intracellular signaling pathway involving STAT3 as key translocation nuclear factor. IL-13 binding to type II receptor complex leads to activation of JAK and phosphorylation of members of the STAT family. The IL-17 receptor complex, which consists of IL-17RA and IL-17RC, activates downstream signaling pathways by recruiting an adaptor molecule named Act1. IL-33 triggers MAPK and NF-κB leading to inflammatory response.

IL-10, one of the major anti-inflammatory cytokines, controls neutrophil infiltration and suppresses various pro-inflammatory mediators (Louis et al., 1998; Sziksz et al., 2020). IL-10 can be produced by Th2 cells but also by Th1, Th17, Tregs, CD8^+^ T, and B lymphocytes. In the liver, IL-10 is expressed in various cell types, including hepatocytes, Kupffer cells, sinusoidal endothelial cells, HSCs, and lymphocytes (Hammerich and Tacke, 2014; Sziksz et al., 2020). Functional IL-10 receptor (IL-10R) complexes are tetramers consisting of two IL-10R1 and two IL-10R2 chains. IL-10 homodimer binding to its receptor activate Janus kinase (JAK) 1 and Tyrosine kinase (TYK) 2. Signal transducer and activator of transcription (STAT) 3 binds to IL-10R1 and phosphorylates, and then phosphorylated STAT3 translocate to the nucleus (Figure 1) (Verma et al., 2016).

### Interleukin-13

IL-13 is an immunoregulatory cytokine secreted by predominantly Th2 cells. IL-13 has multiple effects on the differentiation and function of monocytes and macrophages. Although IL-13 plays an important role in the induction of allergic responses and inflammation as anti-inflammatory cytokine, it is a critical pro-fibrotic factor in liver fibrosis associated with Schistosoma and non-Schistosoma infection (De Vries, 1998; Liu et al., 2012). Additionally, IL-13 has been implicated in inflammatory bowel disease, asthma, and parasitic nematode expulsion (Grunig et al., 1998; Hershey, 2003). IL-13 induces many of the same responses and functional properties as IL-4 and shares a receptor subunit, the α subunit of the IL-4 receptor. IL-13 binds to the complex receptor system comprised of IL-4 receptor and two IL-13 binding proteins, IL-13Rα1 and IL-13Rα2. Signaling through the type II receptor (IL-4 receptor and IL13Rα1) leads to activation of JAK1 or JAK2/TYK2, STAT6, STAT3, and STAT1. Then, STAT dimerization and nuclear translocation occurs, followed by activation of gene transcription (Figure 1) (McCormick and Heller, 2015). IL-13 receptor is expressed on human B cells, basophils, eosinophils, mast cells, endothelial cells, fibroblasts, monocytes, macrophages, respiratory epithelial cells, and smooth muscle cells (Hershey, 2003).

### Interleukin-17

IL-17 (now IL-17A) is an ancient pro-inflammatory cytokine with important roles in defense against bacteria and fungi. IL-17 are produced primarily by a T-cell subset termed Th17 cells, but can also be produced by neutrophils and other lymphocytes (Onishi and Gaffen, 2010). IL-17 signals through the IL-17RA-RC complex, and the IL-17 receptor recruits Act1 for downstream signaling. The Act1 with the IL-17 receptor complex contributes to the recruitment of Tumor necrosis factor receptor associated factor 6 (TRAF6). Then, these kinases promote the production of pro-inflammatory cytokines, chemokines, and-microbial peptides, through the pathway such as nuclear factor-κB (NF-κB), activator protein, and CCAAT/enhancer binding protein pathways (Figure 1) (Onishi and Gaffen, 2010; Meng et al., 2012).

### Interleukin-33

IL-33 is the most recently identified member of IL-1 family of cytokines and is mainly expressed by stromal cells. IL-33 has been shown to induce the Th2 phenotype in Th cell, and therefore promotes progression of Th2-related diseases like several acute and chronic inflammatory diseases, including asthma, atopic dermatitis, rheumatoid arthritis, and ulcerative colitis among others (Schmitz et al., 2005; Sanada et al., 2007). The activity of IL-33 is mediated by binding to the heterodimeric ST2/IL-1 receptor associated protein receptor. Afterward, the complex recruits intracellular signaling molecules, including myeloid differentiation primary response 88 (MyD88)/IL-1 receptor-associated kinase/TRAF6, activating NF-κB, as well as extracellular signal regulated kinase (ERK) 1/2, c-Jun N-terminal kinase, p38 and phosphatidylinositol 3-kinase/protein kinase B (Figure 1) (Pinto et al., 2018; Barbier et al., 2019). Recently, IL-33 has gained attention as a new target or as an early alarm of liver fibrogenesis, as Th2 cells are strongly associated with fibrosis progression (Hammerich and Tacke, 2014; Weiskirchen and Tacke, 2016).

## Contribution of These Target Interleukins in the Hepatic Cells

### Interleukins in Hepatocytes

Hepatocytes are the major parenchymal cells that are important for liver function in metabolism, detoxification, and alcohol processing, and protein synthesis. Hepatocytes also activate innate and adaptive immunity when they are damaged by hepatotropic virus, an intracellular bacterium, or repeated or continuous injury (Crispe, 2016; Zhou et al., 2016). The initial reaction of hepatocytes injury by multiple factors is cell stress and cell death. The stressed and dying hepatocytes release damage-associated molecular patterns (DAMPs), reactive oxygen species, pro-inflammatory signals, and proliferation-associated cytokines through cross-talk with surrounding cells such as HSCs, endothelial cells, and immune cells (Tu et al., 2015; Yan et al., 2018). The reaction of hepatocytes indicate that hepatocytes contribute to the progress and resolution of liver fibrosis as active participants, not victims or bystanders (Tu et al., 2015).

McHedlidze et al. showed that damaged hepatocytes secreted IL-33 and that extracellular IL-33 leads to accumulation and activation of innate lymphoid cells (ILC2) (McHedlidze et al., 2013). Activated ILC2 by IL-33 produce IL-13, which induced the activation and trans-differentiation of HSCs through type-II IL-4 receptor-dependent signaling and STAT6. Further studies have identified IL-33 as key mediator of hepatic fibrosis by demonstrating that IL-33 deficiency ameliorates liver fibrosis induced by bile duct ligation and carbon tetrachloride (CCl_4_) (McHedlidze et al., 2013).

Hepatocytes produce IL-10 which downregulates pro-inflammatory responses and has a potential modulatory effect on liver fibrosis (Sziksz et al., 2020). IL-10 gene therapy attenuated hepatic fibrosis and prevented cell apoptosis in a thioacetamide-treated liver. IL-10 gene transfer reduced not only mRNA level of liver TGF-β1, TNF-α, collagen α1, and cell adhesion molecule, but also decreased the activation of α-smooth muscle actin (α-SMA) and cyclooxygenase-2 (Hung et al., 2005). Other studies have shown that the modification with the IL-10 gene on the buffalo rat liver cells, a rat hepatocytes line, decreased a marked ability to stimulate the primary HSCs proliferation and their expression of α-SMA and procollagen type I, and an accelerated apoptosis of the HSCs was induced (Chen et al., 2012). These results suggest that IL-10 gene therapy might be an effective therapeutic reagent for liver fibrosis.

### Interleukins in HSCs

HSCs play a pivotal role in the initiation, perpetuation, and resolution of liver fibrosis. In response to liver injury, quiescent HSCs trans-differentiate into myofibroblasts, and activated HSCs secrete pro-fibrogenic cytokines, growth factors, and ECM molecules (Higashi et al., 2017). Action of HSCs is also regulated by several soluble factors such as TGFβ, platelet-derived growth factor (PDGF), IL-1β, and interleukins. These soluble factors are derived from hepatocytes, macrophages, and other immune cells. In the case of interleukins, IL-17, IL-13, and IL-33 promote hepatic fibrogenesis through activation of hepatic stellate cells. In contrast, IL-10 and IL-22 protect from development of fibrosis by suppressing pro-fibrogenic function of HSCs (Hammerich and Tacke, 2014).

Many studies demonstrated that IL-13 is the pre-dominant profibrotic Th2 cytokine in Schistosomiasis infection which leads to granuloma formation and subsequent fibrosis development in the liver. Also, they show that blockade of IL-13 prevented liver fibrogenesis (Chiaramonte et al., 1999; Fallon et al., 2000; Chiaramonte et al., 2001). In the liver, IL-13 directly induces expression of collagen I and other critical fibrosis-associated genes such as α-SMA and connective tissue growth factor (CTGF) in HSCs (Liu et al., 2012). In HSCs, IL-13 induced CTGF by activating TGF-β-independent activin receptor-like kinase/Smad signaling via the Erk-mitogen-activated protein kinase (MAPK) pathway (Liu et al., 2011). A different study by Sui et al. showed that IL-13 stimulated proliferation of HSCs and secretion of TGF-β and PDGF by activation protein kinase C in LX-2, a cell line of human HSCs (Sui et al., 2018).

Numerous studies show that levels of IL-17 and its receptor increased in response to liver injury. Specially, the pro-inflammatory signaling of IL-17 has been widely studied in HSCs and Kupffer cells (Meng et al., 2012; Tan et al., 2013). Meng et al. demonstrated that IL-17 induced production of collagen type 1 in HSCs through activation of STAT3 signaling pathway. In addition, the author has demonstrated that the activation of fibrin in IL-17-induced HSCs required STAT3 by showing failure to induce collagen-a1 expression in STAT3-deficient HSCs (Meng et al., 2012). A different study showed that pharmacologic inhibition of IL-17-induced ERK1/2 or p38 significantly attenuated HSCs activation and collagen expression (Tan et al., 2013). *In vivo* experiments demonstrated that blocking IL-17 with anti-IL-17A mAb significantly improved liver function and decreased hepatocellular necrosis, pro-inflammatory cytokines, neutrophils and macrophages influx in bile duct ligation-induced liver fibrosis mice (Zhang et al., 2016).

Previous studies have shown that soluble IL-33 increases the secretion of Th2 cytokines such as IL-6, IL-4 and IL-13, which promote HSCs proliferation, TGF-β synthesis and fibrogenesis (Wynn, 2004; Schmitz et al., 2005). Meanwhile, another study showed that activated HSCs with recombinant IL-33 released IL-6, TGF-β, α-SMA, and collagen via MAPK pathways mediated by ERK, c-Jun N-terminal kinase, and p38 protein kinases. Furthermore, the activation of HSCs, liver injury, and inflammatory cell infiltration were reduced in ST2 (IL-33 receptor)-deficient mice (Tan et al., 2017). Marvie et al. identified that IL-33 is upregulated in human and murine fibrosis, and is expressed by HSCs. Activated HSCs was a source of IL-33 that is strongly associated with fibrosis in chronic liver injury (Marvie et al., 2009). These results suggest that IL-33 can play an important role in the cross-talk between HSCs and Th2 cells in liver fibrosis.

The profibrogenic function of HSCs is suppresses by IL-10 which is one of the major anti-inflammatory cytokines. The increase of α-SMA and NF-κB in HSCs was attenuated by ectogenic IL-10 in CCl4-induced liver fibrosis (Zhang et al., 2006). Recently, studies demonstrated that IL-10 induced senescence of activated HSCs via STAT3-p53 pathway to attenuate liver fibrosis (Huang et al., 2020). The therapeutic effects of IL-10 were shown in experiments *in vivo* in which IL-10 gene therapy reduced the expression of fibrosis-related genes including TGF-β, TNF-α, and collagen α1, and also decreased the activation of α-SMA in thioacetamide-induced liver fibrosis (Hung et al., 2005).

### Interleukins in Hepatic Macrophages

Hepatic macrophages consist of Kupffer cells, the largest population of liver resident macrophages, and inflammatory monocytes-derived macrophages (MoMFs) recruited from blood circulation and originated from bone marrow, spleen, and peritoneum. Recruited MoMFs undergo a process of differentiation into M1 (classically activated) or M2 (alternatively activated) species depending on the micro-environment (Van der Heide et al., 2019). Generally, following liver injury by many different factors, the resident Kupffer cells are activated and produce cytokines and chemokines to recruit monocytes and neutrophils. The recruited Ly6C+ MoMFs release pro-fibrotic molecules like TGF-β, TNF-α, IL-1β, PDGF and CC chemokine ligand 2, and these cytokines activate HSCs and promote liver fibrosis (Dong et al., 2019; Van der Heide et al., 2019). Recently, novel methods on targeting liver macrophages in fibrosis focus on regulating the activation of Kupffer cells, MoMFs recruitment, and macrophage polarization and differentiation (Tacke, 2017).

Kupffer cells not only express the receptor of IL-17, but also produce IL-17 which is mainly produced by Th17 cells (Ge and You, 2008). Meng et al. demonstrated that Kupffer cells, stimulated by IL-17, expressed inflammatory cytokines IL-6, IL-1β, TNF-α, and TGF-β1, which in turn, induced trans-differentiation of HSCs into fibrogenic myofibroblasts, and further facilitated differentiation of IL-17 producing cells (Meng et al., 2012). A very recent study showed that IL-17 is a cytokine that promotes tumors, critically controlling the inflammatory response in macrophages and the cholesterol synthesis in steatotic hepatocytes in the experimental model of alcohol-induced hepatocellular carcinoma. Therefore, the authors suggested the possibility of IL-17 as a potential treatment target for patients with alcohol-induced hepatocellular carcinoma as well as fibrosis (Ma et al., 2020).

Kupffer cells provide an anti-inflammatory microenvironment by secreting the anti-inflammatory cytokines IL-10 (Heymann et al., 2015; Van der Heide et al., 2019). Antigen presentation to Kupffer cells induces the arrest of CD4 T cells and the secretion of IL-10, an immunosuppressive cytokine, which results in promoting the immune tolerance (Van der Heide et al., 2019). Alternatively, activated M2 macrophages inhibit inflammatory reactions and secrete IL-10, IL-4/IL-13, TGF-β, and vascular endothelial growth factor-α to facilitate tissue repair. In contrast to the M2, M1 is pro-inflammatory, microbicidal, and tumoricidal. M1 also releases numerous inflammatory cytokines e.g., TNF-α, IL-1, IL-6, IL-12, IL-15, and IL-18 (Roszer, 2015; Wang et al., 2018). IL-10 mRNA was upregulated from both Kupffer cells *in vitro* and in whole liver after treatment with lipopolysaccharide or CCl4. Also, IL-10 inhibited production of superoxide and TNF-α in rat Kupffer cells following lipopolysaccharide treatment. Furthermore, IL-10−/− mice showed significantly more severe fibrosis than wild type controls after 70 days of injection with CCl4. The authors concluded that synthesized IL-10 may modulate Kupffer cells action during the liver inflammation and fibrosis, and influence subsequent progression of fibrosis (Thompson et al., 1998).

## Discussion

In this review, we have shown the main roles of interleukins in a complex and active cross-talk between hepatic cells responses during liver injury, and their potential as therapeutic targets. Fibrosis is characterized by abnormal ECM derived from HSCs, but it is a dynamic process driven by cytokine-mediated signaling pathways, starting with hepatocytes which are directly involved in the initiation and progression of fibrosis, continuing with macrophages that promote inflammation and present antigens to CD4 T cells which then adjust the immune responses. Interleukins are an immunomodulatory cytokine that is deeply involved from the initiation to the resolution of fibrosis. Recently, the development of a targeted therapy related to liver fibrosis using monoclonal antibodies against interleukins has received considerable interest (Table 1). This antibody treatment targeting interleukins has shown potential as a therapeutic agent in various liver diseases through animal experiments. Treatment with monoclonal antibody neutralizing IL-1β and IL-1 receptor antagonist have been tested in clinical trials. While the anti-cytokine therapies can prevent the progression of hepatic fibrogenesis in the early stages of liver injury, they are not a cure for advanced liver fibrosis. Therefore, treatment at the liver fibrosis stage that prevents the progression of the disease, and that leads to resolution is very important. In addition, hepatic fibrosis treatment strategies using various fusion proteins are detailed in Table 1. Targeting IL-22 is particularly promising as an alternative strategy to directly break the cycle of inflammatory cytokine and chemokine signaling, and the fusion protein using the IL-22 gene and nanocomplex is considered a new strategy to improve liver disease (Chen et al., 2017; Chen et al., 2018; Chen et al., 2020).

**TABLE 1:** Strategies targeting interleukins for liver disease.

Agent	Condition or disease	Objective or effector function	Trial number	References
Antibodies				
Monoclonal antibody neutralizing IL-1β (Canakinumab)	Alcoholic hepatitis	Explore the potential benefits of the IL-1β antibody (Canakinumab) in the treatment of alcoholic hepatitis	NCT03775109	Dinarello et al. (2012)
IL-1 receptor antagonist (Anakinra)	Alcoholic hepatitis	Determine the clinical efficacy and safety of IL-1 receptor antagonist (Anakinra, plus zinc) in participants with clinically severe alcoholic hepatitis	NCT04072822	Meier et al. (2019), Dasarathy et al. (2020)
Polymorphism of IL-1β and TNF-α	Hepatitis B, HCC, Chronic liver disease	Find the effects of polymorphism of IL-1β and TNF-α and their interaction on susceptibility and severity of HBV-related HCC	NCT00629486	Dondeti et al. (2016)
Anti-IL-20 or IL-20R1 monoclonal antibody	Short-term and long-term CCl4 -induced liver injury	Attenuated hepatocyte damage, inhibited TGF-β1 production, liver fibrosis, HSC activation, and ECM accumulation	—	Chiu et al. (2014)
Monoclonal antibody neutralizing IL-11 and anti-IL11RA	Non-alcoholic steatohepatitis	Prevents liver inflammation and steatosis, reverses severe hepatocyte damage, reduces hepatic immune cells and TGFβ1 levels	—	Widjaja et al. (2019)
Fusion protein				
Nanocomplexes with IL-22 gene	Acetaminophen-induced liver injury, Concanavalin A-induced hepatitis. NAFLD	Activated STAT3/Erk signaling, inhibition of reactive oxygen species generation, ameliorate acetaminophen-induced liver injury	—	Chen et al. (2017), Chen et al. (2018), Zai et al. (2019)
Fusion protein of IL-6 and the soluble IL-6 receptor	D-galactosamine induced acute liver injury	Reversed the state of hepatotoxicity, stimulated liver regeneration	—	Galun et al. (2000)
Fusion protein of IL-28B and human serum albumin	Cell culture-derived hepatitis C virus	Inhibited hepatitis C virus infection	—	Fayad et al. (2013)
Fusion protein of IL-13 cytotoxin	Nonalcoholic steatohepatitis	Decline in fibrosis and liver enzymes without organ toxicity, ameliorates pathological features of NASH	—	Shimamura et al. (2008)

CCl4, carbon tetrachloride; ECM, extracellular matrix; HBV, hepatitis B virus; HCC, hepatocellular carcinoma; HSC, hepatic stellate cells; IL, interleukin; NAFLD, nonalcoholic fatty liver disease; STAT3/Erk, signal transducer and activator of transcription 3/extracellular signal regulated kinase; TGF, tumor growth factor; TNF, tumor necrosis factor.

**FIGURE 2:**
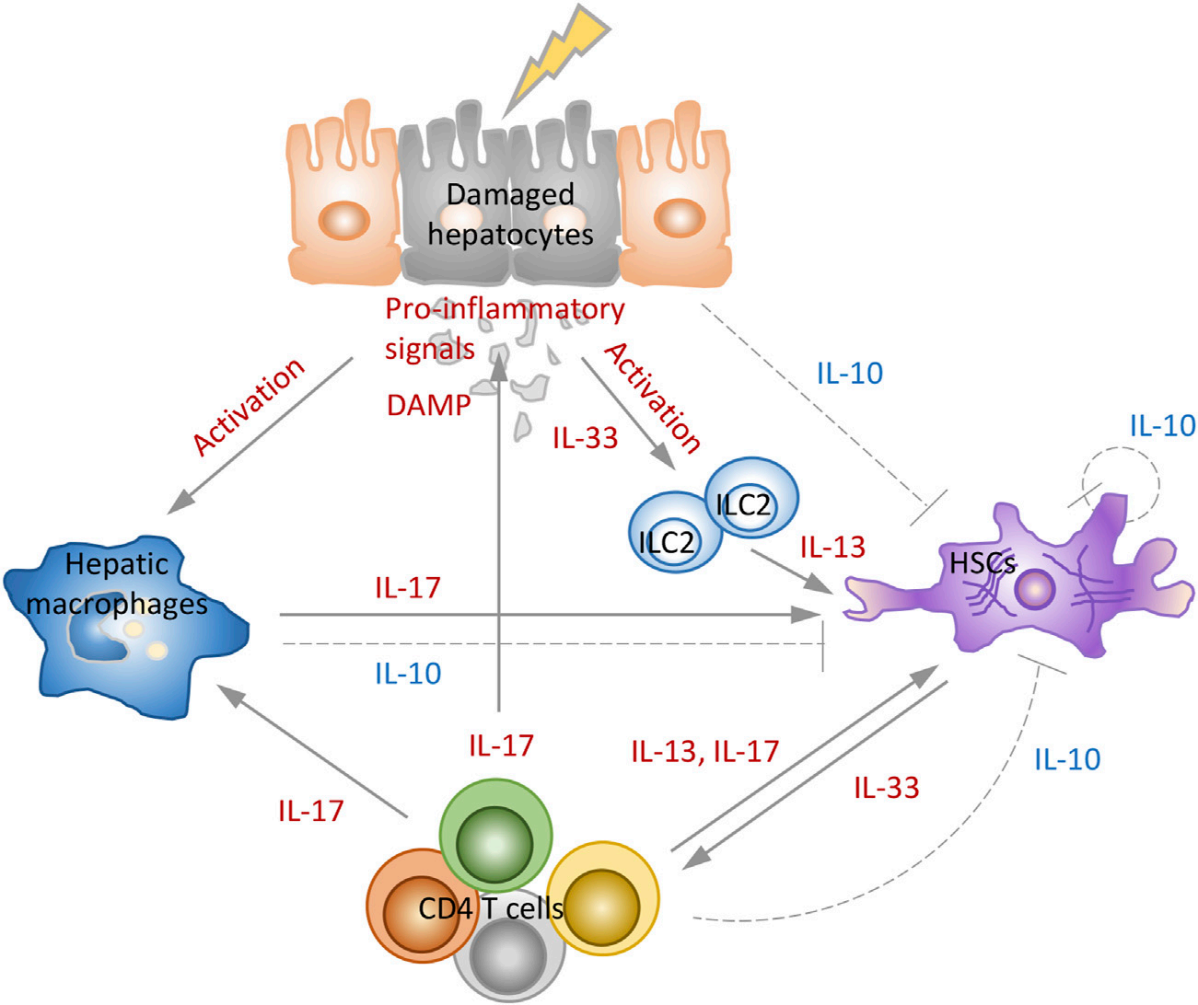
Role of interleukins in development and resolution of liver fibrosis. The combination of signals from inflammatory environment, hepatocytes death, activated macrophages and CD4 T cells stimulates HSCs to proliferate and synthesize collagen and induce fibrosis. IL-33 induces the production of IL-13, and IL-13 and IL-17 directly mediate the activation of HSCs. In the model of fibrosis resolution, hepatocytes, macrophages, HSCs, and CD4 T cells activate the negative feedback loops that reduce immune-mediated fibrosis by secreting IL-10 that prevent activation of HSCs and production of collagen.

Damaged hepatocytes activate hepatic macrophages, and release DAMPs and IL-33. ILC2, which is activated by IL-33 released by hepatocytes, secretes IL-13, that can induce the activity of HSCs. Stimulated macrophages modulate antigen-presentation of CD4 T cells and produce IL-17 together with CD4 T cells, directly affecting the activity of HSCs and collagen production. Additionally, CD4 cells can promote fibrosis by stimulating macrophages and HSCs with IL-17 or IL-13. The quiescent HSCs receiving various pro-inflammatory signals not only transdifferentiate into myofibroblasts to produce collagen, but secrete pro-fibrogenic cytokines such as IL-33 to play pivotal role in fibrosis. On the other hand, during fibrosis resolution, these cells reverse myofibroblast activation with IL-10 and regulate the restoration of homeostasis (Figure 2). This suggests that strategies using prevention of pro-inflammatory interleukins or induction of anti-inflammatory interleukins for the treatment of liver fibrosis may be effective.

IL-3, IL-17 and IL-33 induce liver fibrosis through various mechanisms, therefore an approach targeting them as a major participant in the “fibrosis pathway” is expected to be worthwhile. The blockade of IL-13 can be reversed to reduce liver pathology even when fibrosis is already established. IL-17 signals contribute to the pathogenesis of liver damage and IL-17 inhibition can potentially be an effective treatment for liver disease. Furthermore, IL-17 may be a treatment target for alcohol-induced liver damage and hepatocellular carcinoma. An approach aimed at the IL-33/ST2 path could be a potential therapeutic target for human patients suffering from chronic hepatitis and liver fibrosis (Hammerich and Tacke, 2014; Weiskirchen and Tacke, 2016; Zhang et al., 2016; Ma et al., 2020). IL-10 is a typical anti-inflammatory cytokine that shows signals of conservation mechanism in multiple organs. Specially in the fibrous liver, IL-10 has been proven to induce resolution of fibrosis, therefore it is suggested as a potential therapeutic target (Steen et al., 2020). However, the half-life of recombinant IL-10 *in vivo* is relatively short, and non-targeted administration can cause systemic side effects (Chen et al., 2012). Therefore, increasing numbers of studies have aimed to use IL-10 gene transfer and IL-10 gene therapy might be an effective treatment for liver fibrosis.

Fibrosis can occur in almost any organ or tissue, including lung, heart, and intestine, and is associated with a variety of diseases. Although liver fibrosis has been addressed in this review, treatment with specific interleukins can be applied to several tissue fibrosis. Table 2 shows studies using interleukin 10, 13, 17, and 33 in various fibrotic tissues (Ng et al., 2018; Wijsenbeek et al., 2018; Sziksz et al., 2020). However, there are some practical problems such as the relevant doses of recombinant interleukins, the route of administration, and the costs of production of the cytokine itself, that need to be addressed to move from the experimental stage to the clinical stage. If these hurdles are overcome, a new treatment strategy targeting specific interleukins will be able to deliver expected therapeutic effects not only in the liver, but also in other tissues as well.

**TABLE 2:** Roles of IL-10, 13, 17, and 33 in fibrotic disease.

Interleukin	Producing cell	Receptor	Liver	Lung	Heart	Intestine
IL-10	Basophils, B cells, dendritic cells, eosinophils, neutrophils, macrophages Mast cells, Th2 cells	IL-10R1(a), IL-10R2 (b)	IL-10 inhibited HSCs activation Louis et al. (1998) IL-gene therapy reduced the expression of profibrotic genes Hung et al. (2005) IL-10 KO mice showed more severe liver fibrosis Thompson et al. (1998)	IL-10 KO mice increased inflammation after intratracheal instillation of silica Huaux et al. (1998) genetic delivery of IL-10 attenuated the TGF-β production Nakagome et al. (2006)	The lack of IL-10 resulted more severe myocardial fibrosis Verma et al. (2012) the administration of rIL-10 improved cardiac remodeling Krishnamurthy et al. (2009) IL-10 treatment decreased the myocardial inflammation in mice with autoimmune myocarditis Zimmermann et al. (2012)	Loss of function mutations in the gene of IL-10 caused early onset of IBD Kotlarz et al. (2012) IL-10 supplementation did not result clinical improvements in CD patients Marlow et al. (2013)
IL-13	Basophils, B cells, endothelial cells, eosinophils, epithelial cells, fibroblasts, mast cells, macrophages, monocytes, smooth muscle cells, Th2 cells	IL-4Rα, IL13Rα1	Blockade of IL-13 prevented liver fibrogenesis Chiaramonte et al. (2001) IL-13 induced production of collagen, α-SMA Chiaramonte et al. (1999)	IL-13−/− and IL-4/13−/− mice were protected from lung fibrosis development in response to FITC inoculation Kolodsick et al. (2004) IL-13 and IL-4 are elevated in the bronchial alveolar lavage fluid of IPF patients Park et al. (2009)	Il13Rα1-deficient mice develop severe myocardial dysfunction Amit et al. (2017) deficiency of IL-13 leads to increased leukocyte infiltration and reduced M2-like differentiation of the monocytes in the myocardium Hofmann et al. (2014)	IL-13 production by type 2 NKT cells demonstrated to be critical for colitis development Heller et al. (2002) IL-13 is not increased in fibrotic CD muscle layer Vainer et al. (2000)
IL-17	B cells, dendritic cells, macrophages, Th17 cells	IL-17RA, IL-17RC	The increased level of IL-17 activated HSCs and induced collagen production Meng et al. (2012) elevated levels of IL-17 were also found in the fibrotic livers of patients with hepatitis B virus and cirrhosis related liver damage Du et al. (2013)	Anti-IL-17A neutralizing antibody attenuated pulmonary fibrosis and ECM deposition Chen et al. (2014) in humans, elevated levels of IL-17 and IL-1β were seen in the BAL fluid of patients with IPE Wilson et al. (2010)	IL-17 directly induced VA *in vivo* and *in vitro* in a dose-dependent manner Chang et al. (2018) IL-17 induced cardiac fibrosis both *in vitro* and *in vivo* via PKCβ/Erk1/2/NF-κB signaling pathway Liu et al. (2012)	IL-17 induced HSP47 as well as type I collagen in human intestinal myofibroblasts Honzawa et al. (2014) IL-17 contributed significantly for stricture development in CD Yagi et al. (2007) level of fecal IL-17 was elevated in patients with active CD Biancheri et al. (2013)
IL-33	Basophils, B cells, CD8+T cells, dendritic cells, eosinophils, ILC2s, macrophages, mast cells, natural killer cells, Th2 cells, Tregs cells	ST2, IL1RAcP	IL-33−/− mice showed decrease in collagen deposition and ECM-related gene expression McHedlidze et al. (2013) production of IL-13 in Th2 cells Marvie et al. (2009) IL-33 exacerbated liver fibrosis in mice Gao et al. (2016)	Level of IL-33 was elevated in the bronchoalveolar lavage fluids of patients with IPF Lee et al. (2017) treatment with anti-IL-33 antibody markedly reduced airway inflammation and lung fibrosis Luzina et al. (2013)	Recombinant IL-33 reduced aortic atherosclerotic plaque development Miller et al. (2008) ST2−/− mice showed more cardiac fibrosis and impaired survival Sanada et al. (2007) expression levels of IL-33/ST2 in human myocardial tissue were associated with cardiac fibrosis Tseng et al. (2017)	Inhibition of endogenous ST2-mediated signaling by treatment with neutralizing antibody improved DSS-induced colitis Sedhom et al. (2013) IL-33 has extenuating effects in chronic DSS-induced colitis Grobeta et al. (2012)

BAL, bronchoalveolar lavage; CD, crohn’s disease; DSS, dextran sulfate sodium; ECM, extracellular matrix; Erk, extracellular signal regulated kinase; HSCs, hepatic stellate cells; HSP, heat shock protein; IBD, inflammatory bowel diseases; IL, interleukin; IPF, idiopathic pulmonary fibrosis; KO, knock-out; NF-κb, nuclear factor-κb; NKT, natural killer T cells; PCK, protein kinase C; r, recombinant; SMA, smooth muscle actin; TGF, tumor growth factor; VA, ventricular arrhythmia.

## Abbreviations

α-SMA, α-smooth muscle actin; CCl4, Carbon tetrachloride; CD4 T cells; CD4+ T helper cells; DAMP, Danger associated molecular patterns; ECM, Extracellular matrix; ERK, Extracellular signal regulated kinase; HSCs, Hepatic stellate cells; IL, Interleukin; IL-10R, IL-10 receptor; ILC2, Innate lymphoid cells; JAK, Janus kinase; MAPK; Mitogen-activated protein kinase; MCs, Mast cells; MoMFs, Monocytes-derived macrophages; MyD88, Myeloid differentiation primary response gene 88; NF-κB; Nuclear factor-κB, PDGF, Platelet-derived growth factor; STAT, Signal transducer and activator of transcription; TGF-β, Transforming growth factor-β; Th, T helper; TRAF6, Tumor necrosis factor receptor-associated factor 6; Tregs, Regulatory T cells; TYK, Tyrosine kinase.

## References

[B1] Amit U. Kain D. Wagner A. Sahu A. Nevo-Caspi Y. Gonen N. (2017). New role for interleukin-13 receptor α1 in myocardial homeostasis and heart failure. J. Am. Heart Assoc. 6 (5), e005108. 10.1161/JAHA.116.005108 28528324 PMC5524075

[B2] Bansal R. Nagórniewicz B. Prakash J. (2016). Clinical advancements in the targeted therapies against liver fibrosis. Mediators Inflamm. 2016, 7629724. 10.1155/2016/7629724 27999454 PMC5143744

[B3] Barbier L. Ferhat M. Salamé E. Robin A. Herbelin A. Gombert J. M. (2019). Interleukin-1 family cytokines: keystones in liver inflammatory diseases. Front. Immunol. 10, 2014. 10.3389/fimmu.2019.02014 31507607 PMC6718562

[B4] Bataller R. Brenner D. A. (2005). Liver fibrosis. J. Clin. Invest. 115, 209–218. 10.1172/JCI24282 15690074 PMC546435

[B5] Biancheri P. Pender S. L. Ammoscato F. Giuffrida P. Sampietro G. Ardizzone S. (2013). The role of interleukin 17 in Crohn’s disease-associated intestinal fibrosis. Fibrogenesis Tissue Repair 6, 13. 10.1186/1755-1536-6-13 23834907 PMC3733737

[B6] Caraffa A. Gallenga C. E. Kritas S. K. Ronconi G. Di Emidio P. Conti P. (2019). CAR-T cell therapy causes inflammation by IL-1 which activates inflammatory cytokine mast cells: anti-inflammatory role of IL-37. J. Biol. Regul. Homeost Agents 33, 1981–1985. 10.23812/EditorialCaraffa 31920059

[B7] Chan A. H. Schroder K. (2020). Inflammasome signaling and regulation of interleukin-1 family cytokines. J. Exp. Med. 217, e20190314. 10.1084/jem.20190314 31611248 PMC7037238

[B8] Chang S. L. Hsiao Y. W. Tsai Y. N. Lin S. F. Liu S. H. Lin Y. J. (2018). Interleukin-17 enhances cardiac ventricular remodeling via activating MAPK pathway in ischemic heart failure. J. Mol. Cell. Cardiol. 122, 69–79. 10.1016/j.yjmcc.2018.08.005 30096409

[B9] Chen W. Zai W. Fan J. Zhang X. Zeng X. Luan J. (2020). Interleukin-22 drives a metabolic adaptive reprogramming to maintain mitochondrial fitness and treat liver injury. Theranostics 10, 5879–5894. 10.7150/thno.43894 32483425 PMC7254999

[B10] Chen W. Zhang X. Fan J. Zai W. Luan J. Li Y. (2017). Tethering interleukin-22 to apolipoprotein A-I ameliorates mice from acetaminophen-induced liver injury. Theranostics 7, 4135–4148. 10.7150/thno.20955 29158815 PMC5695002

[B11] Chen W. Luan J. Wei G. Zhang X. Fan J. Zai W. (2018). Vivo hepatocellular expression of interleukin-22 using penetratin-based hybrid nanoparticles as potential anti-hepatitis therapeutics. Biomaterials, 187, 66–80. 10.1016/j.biomaterials.2018.09.046 30296739

[B12] Chen Y. Li C. Weng D. Song L. Tang W. Dai W. (2014). Neutralization of interleukin-17A delays progression of silica-induced lung inflammation and fibrosis in C57BL/6 mice. Toxicol. Appl. Pharmacol. 275, 62–72. 10.1016/j.taap.2013.11.012 24291675

[B13] Chen Y. X. Huang Y. H. Zheng W. D. Chen Z. X. Zhang L. J. Wang X. Z. (2012). Interleukin-10 gene modification attenuates hepatocyte activation of rat hepatic stellate cells *in vitro* . Mol. Med. Rep. 7, 371–378. 10.3892/mmr.2012.1228 23232951

[B14] Chiaramonte M. G. Cheever A. W. Malley J. D. Donaldson D. D. Wynn T. A. (2001). Studies of murine schistosomiasis reveal interleukin-13 blockade as a treatment for established and progressive liver fibrosis. Hepatology 34, 273–282. 10.1053/jhep.2001.26376 11481612

[B15] Chiaramonte M. G. Donaldson D. D. Cheever A. W. Wynn T. A. (1999). An IL-13 inhibitor blocks the development of hepatic fibrosis during a T-helper type 2-dominated inflammatory response. J. Clin. Invest. 104, 777–785. 10.1172/JCI7325 10491413 PMC408441

[B16] Chiu Y. S. Wei C. C. Lin Y. J. Hsu Y. H. Chang M. S. (2014). IL-20 and IL-20R1 antibodies protect against liver fibrosis. Hepatology 60, 1003–1014. 10.1002/hep.27189 24763901

[B17] Crispe I. N. (2016). Hepatocytes as immunological agents. J. Immunol. 196, 17–21. 10.4049/jimmunol.1501668 26685314 PMC4685720

[B18] Dasarathy S. Mitchell M. C. Barton B. Mcclain C. J. Szabo G. Nagy L. E. (2020). Design and rationale of a multicenter defeat alcoholic steatohepatitis trial: (DASH) randomized clinical trial to treat alcohol-associated hepatitis. Contemp. Clin. Trials 96, 106094. 10.1016/j.cct.2020.106094 32739495 PMC7494528

[B19] De Vries J. E. (1998). The role of IL-13 and its receptor in allergy and inflammatory responses. J. Allergy Clin. Immunol. 102, 165–169. 10.1016/s0091-6749(98)70080-6 9723655

[B20] Dinarello C. A. Simon A. Van Der Meer J. W. (2012). Treating inflammation by blocking interleukin-1 in a broad spectrum of diseases. Nat. Rev. Drug Discov. 11, 633–652. 10.1038/nrd3800 22850787 PMC3644509

[B21] Dondeti M. F. El-Maadawy E. A. Talaat R. M. (2016). Hepatitis-related hepatocellular carcinoma: insights into cytokine gene polymorphisms. World J. Gastroenterol. 22, 6800–6816. 10.3748/wjg.v22.i30.6800 27570418 PMC4974580

[B22] Dong X. Liu J. Xu Y. Cao H. (2019). Role of macrophages in experimental liver injury and repair in mice. Exp. Ther. Med. 17, 3835–3847. 10.3892/etm.2019.7450 31007731 PMC6468932

[B23] Du W. J. Zhen J. H. Zeng Z. Q. Zheng Z. M. Xu Y. Qin L. Y. (2013). Expression of interleukin-17 associated with disease progression and liver fibrosis with hepatitis B virus infection: IL-17 in HBV infection. Diagn. Pathol. 8, 40. 10.1186/1746-1596-8-40 23448394 PMC3598543

[B24] Fallon P. G. Richardson E. J. Mckenzie G. J. Mckenzie A. N. (2000). Schistosome infection of transgenic mice defines distinct and contrasting pathogenic roles for IL-4 and IL-13: IL-13 is a profibrotic agent. J. Immunol. 164, 2585–2591. 10.4049/jimmunol.164.5.2585 10679097

[B25] Fayad H. J. Lamare F. Le Rest C. C. Bettinardi V. Visvikis D. (2013). Generation of 4-dimensional CT images based on 4-dimensional PET-derived motion fields. J. Nucl. Med. 54, 631–638. 10.2967/jnumed.112.110809 23471313

[B26] Gallenga C. E. Pandolfi F. Caraffa A. Kritas S. K. Ronconi G. Toniato E. (2019). Interleukin-1 family cytokines and mast cells: activation and inhibition. J. Biol. Regul. Homeost Agents 33, 1–6. 30656901

[B27] Galun E. Zeira E. Pappo O. Peters M. Rose-John S. (2000). Liver regeneration induced by a designer human IL-6/sIL-6R fusion protein reverses severe hepatocellular injury. FASEB J. 14, 1979–1987. 10.1096/fj.99-0913com 11023982

[B28] Gao Y. Liu Y. Yang M. Guo X. Zhang M. Li H. (2016). IL-33 treatment attenuated diet-induced hepatic steatosis but aggravated hepatic fibrosis. Oncotarget 7, 33649–33661. 10.18632/oncotarget.9259 27172901 PMC5085109

[B29] Ge D. You Z. (2008). Expression of interleukin-17RC protein in normal human tissues. Int. Arch. Med. 1, 19. 10.1186/1755-7682-1-19 18928529 PMC2596096

[B30] Groβ P. Doser K. Falk W. Obermeier F. Hofmann C. (2012). IL-33 attenuates development and perpetuation of chronic intestinal inflammation. Inflamm. Bowel Dis. 18, 1900–1909. 10.1002/ibd.22900 22508383

[B31] Grünig G. Warnock M. Wakil A. E. Venkayya R. Brombacher F. Rennick D. M. (1998). Requirement for IL-13 independently of IL-4 in experimental asthma. Science 282, 2261–2263. 10.1126/science.282.5397.2261 9856950 PMC3897229

[B32] Gugliandolo A. Caraffa A. L. Gallenga C. E. Kritas S. K. Ronconi G. Trubiani O. (2019). Mesenchymal stem cells and IL-37: a powerful combination. J. Biol. Regul. Homeost Agents 33, 1019–1022. 31347346

[B33] Hammerich L. Tacke F. (2014). Interleukins in chronic liver disease: lessons learned from experimental mouse models. Clin. Exp. Gastroenterol. 7, 297–306. 10.2147/CEG.S43737 25214799 PMC4158890

[B34] He Y. Hwang S. Ahmed Y. A. Feng D. Li N. Ribeiro M. (2021). Immunopathobiology and therapeutic targets related to cytokines in liver diseases. Cell. Mol. Immunol. 18, 18–37. 10.1038/s41423-020-00580-w 33203939 PMC7853124

[B35] Heller F. Fuss I. J. Nieuwenhuis E. E. Blumberg R. S. Strober W. (2002). Oxazolone colitis, a Th2 colitis model resembling ulcerative colitis, is mediated by IL-13-producing NK-T cells. Immunity 17, 629–638. 10.1016/s1074-7613(02)00453-3 12433369

[B36] Hershey G. K. (2003). IL-13 receptors and signaling pathways: an evolving web. J. Allergy Clin. Immunol. 111, 677–691. 10.1067/mai.2003.1333 12704343

[B37] Heymann F. Peusquens J. Ludwig-Portugall I. Kohlhepp M. Ergen C. Niemietz P. (2015). Liver inflammation abrogates immunological tolerance induced by Kupffer cells. Hepatology 62, 279–291. 10.1002/hep.27793 25810240

[B38] Higashi T. Friedman S. L. Hoshida Y. (2017). Hepatic stellate cells as key target in liver fibrosis. Adv. Drug Deliv. Rev. 121, 27–42. 10.1016/j.addr.2017.05.007 28506744 PMC5682243

[B39] Hofmann U. Knorr S. Vogel B. Weirather J. Frey A. Ertl G. (2014). Interleukin-13 deficiency aggravates healing and remodeling in male mice after experimental myocardial infarction. Circ. Heart Fail. 7, 822–830. 10.1161/CIRCHEARTFAILURE.113.001020 24970469

[B40] Honzawa Y. Nakase H. Shiokawa M. Yoshino T. Imaeda H. Matsuura M. (2014). Involvement of interleukin-17A-induced expression of heat shock protein 47 in intestinal fibrosis in Crohn’s disease. Gut 63, 1902–1912. 10.1136/gutjnl-2013-305632 24534724

[B41] Huang Y. H. Chen M. H. Guo Q. L. Chen Z. X. Chen Q. D. Wang X. Z. (2020). Interleukin-10 induces senescence of activated hepatic stellate cells via STAT3-p53 pathway to attenuate liver fibrosis. Cell. Signal. 66, 109445. 10.1016/j.cellsig.2019.109445 31730896

[B42] Huaux F. Louahed J. Hudspith B. Meredith C. Delos M. Renauld J. C. (1998). Role of interleukin-10 in the lung response to silica in mice. Am. J. Respir. Cell Mol. Biol. 18, 51–59. 10.1165/ajrcmb.18.1.2911 9448045

[B43] Hung K. S. Lee T. H. Chou W. Y. Wu C. L. Cho C. L. Lu C. N. (2005). Interleukin-10 gene therapy reverses thioacetamide-induced liver fibrosis in mice. Biochem. Biophys. Res. Commun. 336, 324–331. 10.1016/j.bbrc.2005.08.085 16126171

[B44] Kagan P. Sultan M. Tachlytski I. Safran M. Ben-Ari Z. (2017). Both MAPK and STAT3 signal transduction pathways are necessary for IL-6-dependent hepatic stellate cells activation. PLoS One 12, e0176173. 10.1371/journal.pone.0176173 28472150 PMC5417441

[B45] Kolodsick J. E. Toews G. B. Jakubzick C. Hogaboam C. Moore T. A. Mckenzie A. (2004). Protection from fluorescein isothiocyanate-induced fibrosis in IL-13-deficient, but not IL-4-deficient, mice results from impaired collagen synthesis by fibroblasts. J. Immunol. 172, 4068–4076. 10.4049/jimmunol.172.7.4068 15034018

[B46] Kotlarz D. Beier R. Murugan D. Diestelhorst J. Jensen O. Boztug K. (2012). Loss of interleukin-10 signaling and infantile inflammatory bowel disease: implications for diagnosis and therapy. Gastroenterology 143, 347–355. 10.1053/j.gastro.2012.04.045 22549091

[B47] Krishnamurthy P. Rajasingh J. Lambers E. Qin G. Losordo D. W. Kishore R. (2009). IL-10 inhibits inflammation and attenuates left ventricular remodeling after myocardial infarction via activation of STAT3 and suppression of HuR. Circ. Res. 104, e9–18. 10.1161/CIRCRESAHA.108.188243 19096025 PMC2774810

[B48] Kubes P. Jenne C. (2018). Immune responses in the liver. Annu. Rev. Immunol. 36, 247–277. 10.1146/annurev-immunol-051116-052415 29328785

[B49] Lee J. U. Chang H. S. Lee H. J. Jung C. A. Bae D. J. Song H. J. (2017). Upregulation of interleukin-33 and thymic stromal lymphopoietin levels in the lungs of idiopathic pulmonary fibrosis. BMC Pulm. Med. 17, 39. 10.1186/s12890-017-0380-z 28202030 PMC5312598

[B50] Li S. Tan H. Y. Wang N. Feng Y. Wang X. Feng Y. (2019). Recent insights into the role of immune cells in alcoholic liver disease. Front. Immunol. 10, 1328. 10.3389/fimmu.2019.01328 31244862 PMC6581703

[B51] Liu Y. Meyer C. Müller A. Herweck F. Li Q. Müllenbach R. (2011). IL-13 induces connective tissue growth factor in rat hepatic stellate cells via TGF-β-independent Smad signaling. J. Immunol. 187, 2814–2823. 10.4049/jimmunol.1003260 21804025

[B52] Liu Y. Munker S. Müllenbach R. Weng H. L. (2012). IL-13 signaling in liver fibrogenesis. Front. Immunol. 3, 116. 10.3389/fimmu.2012.00116 22593760 PMC3349963

[B53] Lokau J. Schoeder V. Haybaeck J. Garbers C. (2019). Jak-stat signaling induced by interleukin-6 family cytokines in hepatocellular carcinoma. Cancers (Basel) 11, 1704. 10.3390/cancers11111704 PMC6896168 31683891

[B54] Louis H. Van Laethem J. L. Wu W. Quertinmont E. Degraef C. Van Den Berg K. (1998). Interleukin-10 controls neutrophilic infiltration, hepatocyte proliferation, and liver fibrosis induced by carbon tetrachloride in mice. Hepatology 28, 1607–1615. 10.1002/hep.510280621 9828225

[B55] Luckheeram R. V. Zhou R. Verma A. D. Xia B. (2012). CD4⁺T cells: differentiation and functions. Clin. Dev. Immunol. 2012, 925135. 10.1155/2012/925135 22474485 PMC3312336

[B56] Luzina I. G. Kopach P. Lockatell V. Kang P. H. Nagarsekar A. Burke A. P. (2013). Interleukin-33 potentiates bleomycin-induced lung injury. Am. J. Respir. Cell Mol. Biol. 49, 999–1008. 10.1165/rcmb.2013-0093OC 23837438 PMC3931117

[B57] Ma H. Y. Yamamoto G. Xu J. Liu X. Karin D. Kim J. Y. (2020). IL-17 signaling in steatotic hepatocytes and macrophages promotes hepatocellular carcinoma in alcohol-related liver disease. J. Hepatol. 72, 946–959. 10.1016/j.jhep.2019.12.016 31899206 PMC7167339

[B58] Mair M. Zollner G. Schneller D. Musteanu M. Fickert P. Gumhold J. (2010). Signal transducer and activator of transcription 3 protects from liver injury and fibrosis in a mouse model of sclerosing cholangitis. Gastroenterology 138, 2499–2508. 10.1053/j.gastro.2010.02.049 20193684

[B59] Marlow G. J. Van Gent D. Ferguson L. R. (2013). Why interleukin-10 supplementation does not work in Crohn’s disease patients. World J. Gastroenterol. 19, 3931–3941. 10.3748/wjg.v19.i25.3931 23840137 PMC3703179

[B60] Marvie P. Lisbonne M. L’helgoualc’h A. Rauch M. Turlin B. Preisser L. (2009). Interleukin-33 overexpression is associated with liver fibrosis in mice and humans. J. Cell Mol. Med. 14, 1726–1739. 10.1111/j.1582-4934.2009.00801.x 19508382 PMC3829034

[B61] McCormick S. M. Heller N. M. (2015). Commentary: IL-4 and IL-13 receptors and signaling. Cytokine 75, 38–50. 10.1016/j.cyto.2015.05.023 26187331 PMC4546937

[B62] McHedlidze T. Waldner M. Zopf S. Walker J. Rankin A. L. Schuchmann M. (2013). Interleukin-33-dependent innate lymphoid cells mediate hepatic fibrosis. Immunity 39, 357–371. 10.1016/j.immuni.2013.07.018 23954132 PMC4172965

[B63] Meier R. P. H. Meyer J. Montanari E. Lacotte S. Balaphas A. Muller Y. D. (2019). Interleukin-1 receptor antagonist modulates liver inflammation and fibrosis in mice in a model-dependent manner. Int. J. Mol. Sci. 20 (6), 1295. 10.3390/ijms20061295 PMC6471711 30875826

[B64] Meng F. Wang K. Aoyama T. Grivennikov S. I. Paik Y. Scholten D. (2012). Interleukin-17 signaling in inflammatory, Kupffer cells, and hepatic stellate cells exacerbates liver fibrosis in mice. Gastroenterology 143, 765–776.e3. 10.1053/j.gastro.2012.05.049 22687286 PMC3635475

[B65] Miller A. M. Xu D. Asquith D. L. Denby L. Li Y. Sattar N. (2008). IL-33 reduces the development of atherosclerosis. J. Exp. Med. 205, 339–346. 10.1084/jem.20071868 18268038 PMC2271006

[B66] Nakagome K. Dohi M. Okunishi K. Tanaka R. Miyazaki J. Yamamoto K. (2006). *In vivo* IL-10 gene delivery attenuates bleomycin induced pulmonary fibrosis by inhibiting the production and activation of TGF-beta in the lung. Thorax 61, 886–894. 10.1136/thx.2005.056317 16809410 PMC2104751

[B67] Nanchahal J. Hinz B. (2016). Strategies to overcome the hurdles to treat fibrosis, a major unmet clinical need. Proc. Natl. Acad. Sci. U.S.A. 113, 7291–7293. 10.1073/pnas.1607896113 27342865 PMC4941454

[B68] Ng B. Dong J. D’agostino G. Viswanathan S. Widjaja A. A. Lim W. W. (2018). Interleukin-11 is a therapeutic target in idiopathic pulmonary fibrosis. Sci. Transl Med. 11, eaaw1237. 10.1126/scitranslmed.aaw1237 31554736

[B69] Onishi R. M. Gaffen S. L. (2010). Interleukin-17 and its target genes: mechanisms of interleukin-17 function in disease. Immunology 129, 311–321. 10.1111/j.1365-2567.2009.03240.x 20409152 PMC2826676

[B70] Park S. W. Ahn M. H. Jang H. K. Jang A. S. Kim D. J. Koh E. S. (2009). Interleukin-13 and its receptors in idiopathic interstitial pneumonia: clinical implications for lung function. J. Korean Med. Sci. 24, 614–620. 10.3346/jkms.2009.24.4.614 19654941 PMC2719183

[B71] Paul W. E. Seder R. A. (1994). Lymphocyte responses and cytokines. Cell 76, 241–251. 10.1016/0092-8674(94)90332-8 7904900

[B72] Pinto S. M. Subbannayya Y. Rex D. A. B. Raju R. Chatterjee O. Advani J. (2018). A network map of IL-33 signaling pathway. J. Cell Commun. Signal. 12, 615–624. 10.1007/s12079-018-0464-4 29705949 PMC6039344

[B73] Roszer T. (2015). Understanding the mysterious M2 macrophage through activation markers and effector mechanisms. Mediators Inflamm. 2015, 816460. 10.1155/2015/816460 26089604 PMC4452191

[B74] Sanada S. Hakuno D. Higgins L. J. Schreiter E. R. Mckenzie A. N. Lee R. T. (2007). IL-33 and ST2 comprise a critical biomechanically induced and cardioprotective signaling system. J. Clin. Invest. 117, 1538–1549. 10.1172/JCI30634 17492053 PMC1865027

[B75] Schmidt-Arras D. Rose-John S. (2016). IL-6 pathway in the liver: from physiopathology to therapy. J. Hepatol. 64, 1403–1415. 10.1016/j.jhep.2016.02.004 26867490

[B76] Schmitz J. Owyang A. Oldham E. Song Y. Murphy E. Mcclanahan T. K. (2005). IL-33, an interleukin-1-like cytokine that signals via the IL-1 receptor-related protein ST2 and induces T helper type 2-associated cytokines. Immunity 23, 479–490. 10.1016/j.immuni.2005.09.015 16286016

[B77] Sedhom M. A. Pichery M. Murdoch J. R. Foligné B. Ortega N. Normand S. (2013). Neutralisation of the interleukin-33/ST2 pathway ameliorates experimental colitis through enhancement of mucosal healing in mice. Gut 62, 1714–1723. 10.1136/gutjnl-2011-301785 23172891 PMC3841767

[B78] Shimamura T. Fujisawa T. Husain S. R. Kioi M. Nakajima A. Puri R. K. (2008). Novel role of IL-13 in fibrosis induced by nonalcoholic steatohepatitis and its amelioration by IL-13R-directed cytotoxin in a rat model. J. Immunol. 181, 4656–4665. 10.4049/jimmunol.181.7.4656 18802068

[B79] Steen E. H. Wang X. Balaji S. Butte M. J. Bollyky P. L. Keswani S. G. (2020). The role of the anti-inflammatory cytokine interleukin-10 in tissue fibrosis. Adv. Wound Care (New Rochelle) 9, 184–198. 10.1089/wound.2019.1032 32117582 PMC7047112

[B80] Sui G. Cheng G. Yuan J. Hou X. Kong X. Niu H. (2018). Interleukin (IL)-13, prostaglandin E2 (PGE2), and prostacyclin 2 (PGI2) activate hepatic stellate cells via protein kinase C (PKC) pathway in hepatic fibrosis. Med. Sci. Monit. 24, 2134–2141. 10.12659/msm.906442 29633755 PMC5909417

[B81] Sziksz E. Pap D. Lippai R. Béres N. J. Fekete A. Szabó A. J. (2020). Fibrosis related inflammatory mediators: role of the IL-10 cytokine family. Mediators Inflamm. 2015, 764641. 10.1155/2015/764641 PMC4495231 26199463

[B82] Tacke F. (2017). Targeting hepatic macrophages to treat liver diseases. J. Hepatol. 66, 1300–1312. 10.1016/j.jhep.2017.02.026 28267621

[B83] Tan Z. Liu Q. Jiang R. Lv L. Shoto S. S. Maillet I. (2017). Interleukin-33 drives hepatic fibrosis through activation of hepatic stellate cells. Cell. Mol. Immunol. 15, 388–398. 10.1038/cmi.2016.63 28194023 PMC6052839

[B84] Tan Z. Qian X. Jiang R. Liu Q. Wang Y. Chen C. (2013). IL-17A plays a critical role in the pathogenesis of liver fibrosis through hepatic stellate cell activation. J. Immunol. 191, 1835–1844. 10.4049/jimmunol.1203013 23842754

[B85] Thompson K. Maltby J. Fallowfield J. Mcaulay M. Millward-Sadler H. Sheron N. (1998). Interleukin-10 expression and function in experimental murine liver inflammation and fibrosis. Hepatology 28, 1597–1606. 10.1002/hep.510280620 9828224

[B86] Toniato E. Frydas I. Robuffo I. Ronconi G. Caraffa A. Kritas S. K. (2017). Activation and inhibition of adaptive immune response mediated by mast cells. J. Biol. Regul. Homeost Agents 31, 543–548. 28952282

[B87] Tseng C. C. S. Huibers M. M. H. Van Kuik J. De Weger R. A. Vink A. De Jonge N. (2018). The interleukin-33/ST2 pathway is expressed in the failing human heart and associated with pro-fibrotic remodeling of the myocardium. J. Cardiovasc. Transl Res. 11, 15–21. 10.1007/s12265-017-9775-8 29285671 PMC5846972

[B88] Tu T. Calabro S. R. Lee A. Maczurek A. E. Budzinska M. A. Warner F. J. (2015). Hepatocytes in liver injury: victim, bystander, or accomplice in progressive fibrosis? J. Gastroenterol. Hepatol. 30, 1696–1704. 10.1111/jgh.13065 26239824

[B89] Vainer B. Nielsen O. H. Hendel J. Horn T. Kirman I. (2000). Colonic expression and synthesis of interleukin 13 and interleukin 15 in inflammatory bowel disease. Cytokine 12, 1531–1536. 10.1006/cyto.2000.0744 11023669

[B90] Van der Heide D. Weiskirchen R. Bansal R. (2019). Therapeutic targeting of hepatic macrophages for the treatment of liver diseases. Front. Immunol. 10, 2852. 10.3389/fimmu.2019.02852 31849997 PMC6901832

[B91] Varvara G. Tettamanti L. Gallenga C. E. Caraffa A. D'ovidio C. Mastrangelo F. (2018). Stimulated mast cells release inflammatory cytokines: potential suppression and therapeutical aspects. J. Biol. Regul. Homeost Agents 32, 1355–1360. 30574739

[B92] Verma R. Balakrishnan L. Sharma K. Khan A. A. Advani J. Gowda H. (2016). A network map of Interleukin-10 signaling pathway. J. Cell Commun. Signal. 10, 61–67. 10.1007/s12079-015-0302-x 26253919 PMC4850137

[B93] Verma S. K. Krishnamurthy P. Barefield D. Singh N. Gupta R. Lambers E. (2012). Interleukin-10 treatment attenuates pressure overload-induced hypertrophic remodeling and improves heart function via signal transducers and activators of transcription 3-dependent inhibition of nuclear factor-κB. Circulation 126, 418–429. 10.1161/CIRCULATIONAHA.112.112185 22705886 PMC3422741

[B94] Wang L. X. Zhang S. X. Wu H. J. Rong X. L. Guo J. (2019). M2b macrophage polarization and its roles in diseases. J. Leukoc. Biol. 106, 345–358. 10.1002/JLB.3RU1018-378RR 30576000 PMC7379745

[B95] Weiskirchen R. Tacke F. (2016). Interleukin-33 in the pathogenesis of liver fibrosis: alarming ILC2 and hepatic stellate cells. Cell Mol. Immunol. 14, 143–145. 10.1038/cmi.2016.62 28017959 PMC5301158

[B96] Widjaja A. A. Singh B. K. Adami E. Viswanathan S. Dong J. D'agostino G. A. (2019). Inhibiting interleukin 11 signaling reduces hepatocyte death and liver fibrosis, inflammation, and steatosis in mouse models of nonalcoholic steatohepatitis. Gastroenterology 157, 777–792. 10.1053/j.gastro.2019.05.002 31078624

[B97] Wijsenbeek M. S. Kool M. Cottin V. (2018). Targeting interleukin-13 in idiopathic pulmonary fibrosis: from promising path to dead end. Eur. Respir. J. 52, 1802111. 10.1183/13993003.02111-2018 30545962

[B98] Wilson M. S. Madala S. K. Ramalingam T. R. Gochuico B. R. Rosas I. O. Cheever A. W. (2010). Bleomycin and IL-1beta-mediated pulmonary fibrosis is IL-17A dependent. J. Exp. Med. 207, 535–552. 10.1084/jem.20092121 20176803 PMC2839145

[B99] Wu Y. Min J. Ge C. Shu J. Tian D. Yuan Y. (2020). Interleukin 22 in liver injury, inflammation and cancer. Int. J. Biol. Sci. 16, 2405–2413. 10.7150/ijbs.38925 32760208 PMC7378634

[B100] Wynn T. A. (2004). Fibrotic disease and the T(H)1/T(H)2 paradigm. Nat. Rev. Immunol. 4, 583–594. 10.1038/nri1412 15286725 PMC2702150

[B101] Yagi Y. Andoh A. Inatomi O. Tsujikawa T. Fujiyama Y. (2007). Inflammatory responses induced by interleukin-17 family members in human colonic subepithelial myofibroblasts. J. Gastroenterol. 42, 746–753. 10.1007/s00535-007-2091-3 17876544

[B102] Yan Y. Y. Lin S. Zhu Y. Y. (2018). Damage-associated molecular patterns and liver failure. Zhonghua Gan Zang Bing Za Zhi 24, 636–640. 10.3760/cma.j.issn.1007-3418.2016.08.017 PMC12769654 27788716

[B103] Zai W. Chen W. Wu Z. Jin X. Fan J. Zhang X. (2019). Targeted interleukin-22 gene delivery in the liver by polymetformin and penetratin-based hybrid nanoparticles to treat nonalcoholic fatty liver disease. ACS Appl. Mater. Inter. 11, 4842–4857. 10.1021/acsami.8b19717 30628769

[B104] Zhang L. J. Zheng W. D. Shi M. N. Wang X. Z. (2006). Effects of interleukin-10 on activation and apoptosis of hepatic stellate cells in fibrotic rat liver. World J. Gastroenterol. 12, 1918–1923. 10.3748/wjg.v12.i12.1918 16609999 PMC4087518

[B105] Zhang S. Huang D. Weng J. Huang Y. Liu S. Zhang Q. (2016). Neutralization of interleukin-17 attenuates cholestatic liver fibrosis in mice. Scand. J. Immunol. 83, 102–108. 10.1111/sji.12395 26484852

[B106] Zhou Z. Xu M. J. Gao B. (2016). Hepatocytes: a key cell type for innate immunity. Cell Mol. Immunol. 13, 301–315. 10.1038/cmi.2015.97 26685902 PMC4856808

[B107] Zhu J. Paul W. E. (2008). CD4 T cells: fates, functions, and faults. Blood 112, 1557–1569. 10.1182/blood-2008-05-078154 18725574 PMC2518872

[B108] Zimmermann O. Homann J. M. Bangert A. Müller A. M. Hristov G. Goeser S. (2012). Successful use of mRNA-nucleofection for overexpression of interleukin-10 in murine monocytes/macrophages for anti-inflammatory therapy in a murine model of autoimmune myocarditis. J. Am. Heart Assoc. 1, e003293. 10.1161/JAHA.112.003293 23316321 PMC3540678

